# Advances in drug therapy for systemic lupus erythematosus

**DOI:** 10.1186/1741-7015-8-77

**Published:** 2010-11-29

**Authors:** Daniel J Wallace

**Affiliations:** 1 Division of Rheumatology, Cedars-Sinai Medical Center, 8737 Beverly Blvd, Suite 302, West Hollywood, CA 90048, USA

## Abstract

Systemic lupus erythematosus (SLE) is an autoimmune disorder that afflicts 500,000 people in the United States. There has not been a new SLE drug approved in the United States since 1958. However, a guidance document issued by the Food and Drug Administration in 2005 provided a roadmap for investigators which spawned numerous ongoing clinical trials. Among these, Belimumab, a monoclonal antibody to soluble B lymphocyte stimulator, met its primary endpoints in two large trials and will probably obtain FDA approval soon. Other promising agents targeting a variety of mechanisms of action are currently in development. This minireview highlights the latest therapies under investigation in SLE and gives an overview of the pathways that are specifically being targeted.

## Introduction

Systemic lupus erythematosus (SLE, commonly referred to as lupus) is a pleomorphic, autoimmune disorder of unknown cause. Afflicting approximately 500,000 people in the United States, SLE is characterized by considerable mortality and morbidity. For example, with existing therapies, half of patients with organ-threatening disease (for example, cardiopulmonary, hepatic, renal, central nervous system or autoimmune hemolytic anemia) do not survive 20 years after diagnosis, and the quality of life for those individuals with all forms of SLE is usually seriously compromised [1]. To date, the only agents approved by the Food and Drug Administration (FDA) for SLE are corticosteroids, antimalarials and aspirin, with hydroxychloroquine being the most recent addition to this armamentarium (in 1958). Now, developments in our understanding of the underlying pathogenesis of SLE have led to promising new leads for SLE drug therapy.

SLE drug development was stunted by SLE's being a "woman's disease," inadequate SLE advocacy and mostly by the National Institutes of Health nephritis trial that ran for 20 years in the 1970 s and 1980 s, which demonstrated that a mean time of 5 years elapsed before a treatment arm (intravenous cyclophosphamide versus azathioprine, prednisone alone, or a combination oral cyclophosphamide plus azathioprine) was found to be superior [2]. Not being that patient, the pharmaceutical industry invested its rheumatic disease resources into the rheumatoid arthritis (RA) and spondylitis pipeline, where outcomes were evident within 3-6 months. In 2005, the *Federal Register *published a guidance document (finalized in 2010) aimed at investigators and industry that provided a roadmap detailing what a SLE trial would need to demonstrate to shepherd a new agent to market [3]. This led to the launches of a number of trials. Many errors were made by experienced lupologists (myself included) who were new to the art of clinical trial design. This included giving both arms of a study effective treatment, including patients whose diagnosis of SLE was dubious, overestimation of flare rates resulting in underpowered studies, failure to guarantee adequate supply of a drug, faulty infusion directions, underdosing effective drugs for financial reasons and overestimating steroid requirements. This minireview critically analyzes the current state of the art in SLE drug development from a regulatory and implementation standpoint.

### Ground rules: Requirements for a new SLE drug

The June 2010 FDA guidelines indicate that a candidate SLE drug should meet its primary endpoint in two adequate well-controlled trials demonstrating superiority [4]. Studies should be at least 1 year in duration, and enrollees should fulfill the American College of Rheumatology criteria for SLE. Steroid use variability should be minimized, and sparing effects, if any, should be defined. Study patients should be stratified by the severity of their SLE, with the British Isles Lupus Assessment Group (BILAG) 2004 [5] guidelines being the preferred index for measuring disease reduction (although the Systemic Lupus Erythematosus Disease Activity Index (SLEDAI), European Community Lupus Activity Measure (ECLAM) and Systemic Lupus Activity Measure (SLAM) are also acceptable). The document provides definitions for partial clinical response, remission, reduction in flare and increase in time to flare; encourages the use of patient-reported outcome measures; and leaves the door open for biomarkers and surrogate markers (none of the current ones being acceptable) potentially applicable to shorten the duration of a trial as well as improving our measurement of disease activity. Any agent must demonstrate a satisfactory safety profile, and the document supports the use of organ-specific measures (for example, the Cutaneous Lupus Activity Disease Area and Severity Index (CLASI) for cutaneous disease), especially if the drug is efficacious for one aspect of the disease but not another. The 2010 guidance document takes into account "lessons learned" and nuances that make SLE drug development so complex.

### The use of nontargeted agents: Important recent studies

The overwhelming majority of agents in development are biologics. However, some nonbiological agents and drugs that are on the market for other disorders have been or are under study for SLE. A detailed discussion of these studies is beyond the scope of this minireview, but the salient points are summarized below:

1. Fish oil is ameliorative in patients with mild activity [6].

2. A large trial evaluating the efficacy of vitamin D is in progress (NCT 00418507).

3. The Canadian Cooperative Consortium recently demonstrated that methotrexate is steroid sparing and has anti-inflammatory properties [7].

4. Mycophenolate mofetil is equivalent to cyclophosphamide as induction therapy for SLE nephritis and is superior to azathioprine for maintenance [8,9].

5. Topical pinecrolimus and tacrolimus are effective for chronic cutaneous SLE [10].

6. Leflunomide improves SLE arthritis [11].

7. Dehydroepiandrostrone has modest effects at best in mild SLE and may diminish fatigue and bone demineralization, as well as having steroid sparing properties [12].

In summary, none of the above agents are significantly ameliorative of SLE, and none have been shown to significantly influence its morbidity or mortality when compared to other agents currently available.

### Targeted therapies and SLE: Current status

Multiple mechanisms of action are being explored in a variety of targeted therapies. They are summarized in Table 1.

**Table 1:** Targets for new therapies in systemic lupus erythematosusa

Mechanism of action	Examples of targets
T cells	CTLA4-Ig, modified CD40L, inhibition of ICOS
	Regulatory T cells: expanding CD4+CD25+, CD8+CD28-
B cells	mAbs to CD20, CD22, BlyS, TACi-Ig, BAFF-RFc
	Proteosome/plasma cells
Cytokines	Inhibition of IL-6, IL-10; TNF inhibitors
Innate immune system	Inhibition of IFN-α and IFN-γ, blockade of TLR-7 and/or TLR-9, C5a inhibition
Toleragens	Peptides derived from nucleosomes, splicosomes
Cell surface receptor activation inhibition	Syk kinase, sirolimus

^a^ICOS, ; mAbs, monoclonal antibodies; BlyS, B lymphocyte stimulation; TACi-Ig, immunoglobulin; BAFF-RFc, ; TNF, tumor necrosis factor; IFN, interferon; TLR, Toll-like receptor.

### Immune cell-specific targets (B and T cells)

Inhibition of T cell activation and blockade of the costimulatory pathway is a promising approach (Table 2). Abatacept, already approved for RA, appears to improve musculoskeletal SLE and has an excellent safety profile [13]. The sponsor's choice to frontload their pivotal trial with a forced steroid taper before the agent reached its maximal efficacy was unfortunate and led to the primary endpoint not being met. Bristol-Myers Squibb and the Immune Tolerance Network have nephritis trials in progress, and a new phase III trial for musculoskeletal SLE is planned. Blockade of the CD40L pathway was successful but not safe with BG9588 (because of thrombotic complications) and safe but not effective for IDEC [14,15]. New agents targeting different epitopes which interrupt this pathway are being studied. Amgen 557 is an ICOS-B7-RP1 (Inducible Costimulator B7-RPI are receptor sites) inhibitor under evaluation in a phase II trial. Efalizumab, an LFA-1 inhibitor (Lymphocyte function associated Antigen-1), was withdrawn from the market in 2010 because of concerns with progressive multifocal leukoencephalopathy, and its cutaneous SLE trial was suspended. A phase I safety study of an agent which promotes T regulatory cells (Treg) has been presented [16].

**Table 2:** Important agents currently enrolling patients in clinical trials for systemic lupus erythematosus as of November 2010

Target	Trial	Comment
T cells	NCT007744752	Abatacept
	NCT00774943	Amgen 557 ICOS inhibitor
B cells	NCT00660881	Epratuzumab (anti-CD22)
	NCT00624338	Atacicept blocks BlyS and APRIL
	NCT01162681	A-623 blocks BlyS and APRIL
	NCT01205348	LY2127399 blocks BlyS
Toleragen	NCT01085097	Laquinomod
	NCT01135459	Lupuzor tolerizes splicosome
Innate immunity	NCT00962832	Rontalizumab inhibits IFN-α
	NCT01164917	Amgen 811 targets IFN-γ
	NCT00960362	NNCO152 targets IFN-α
	NCT01031836	MEDI-545 targets IFN-α
Cell surface receptor	NCT0077194	Rapamycin targets mTOR

^a^ICOS, ; BlyS, B lymphocyte stimulation; APRIL, ; mTOR (mammalian target of rapamycin).

B cell depletion via the anti-CD20 rituximab (Roche, Basel, Switzerland) was not successful in EXPLORER or LUNAR "generalized lupus" or nephritis-specific designed studies where all patients were given high doses of corticosteroids and immune suppressives as well [17,18]. Case series clearly suggest that rituximab ameliorates hemolytic anemia, thrombocytopenia, arthritis and probably central nervous system vasculitis associated with SLE. Trials with humanized anti-CD20 s (for example, ocrelizumab (Roche), Trubion's (Trubion, Seattle, WA, USA) small molecule immunopharmaceutical, or SMIP (Small Molecular Immunopharmaceutical); (Pfizer, New York, NY, USA) were halted for economic, safety or design reasons, and none are in progress at this time. Anti-CD22 is a less potent B cell depletor that internalizes cell signaling promoting proinflammatory actions. A phase II dose ranging and safety study with epratuzumab (UNIM Chemique Belge (UCB), Belgium, Brussles) showed significant improvements in BILAG scores, and a phase III trial will start in late 2010 [19].

Belimumab (Human Genome Sciences, (Rockville, MD, USA)/Glaxo Smith Kline, (Uxbridge, UK) is a fully human monoclonal antibody that selectively targets and inhibits soluble B lymphocyte stimulation (BLyS), resulting in autoreactive B cell apoptosis. Using an FDA-endorsed responder index that includes improvement in the Systematic Lupus Erythematosus Disease Activity Scale (SLEDAI) score, no new BILAG organ system occurrences and no worsening in physician assessments, phase II and phase III trials significantly met this endpoint among over 3,000 treated patients that resulted in an application for approval to the FDA in June 2010 [20-22]. BLyS and A Proliferatiave Inducing Ligand (APRIL) are ligands for receptors BAFF-R (B Cell Activation Factor), BCMA (B Cell Maturation Associate) and TACI(Transmembrane Activator and Calcium Reproducing Initiator). Agents blocking these components in addition to BlyS (or inhibiting membrane BlyS in addition to soluble BlyS) are being studied. These include LY2127399 (Lilly), atacicept (in phase III trials; Merck-Serono), BR-3Fc blockade (Biogen, Cambridge, MA, USA) and A-623 (Amgen/Anthera, Hayward, CA, USa). As with belimumab, these drugs primarily target developing B cells and have minimal actions on the bone marrow or plasma cells. Bortezomib and carfilzumib are small-molecule inhibitors used for multiple myeloma. These agents block plasma cells, and SLE clinical trials are on the drawing board.

In summary, agents which block the action of T and B cells are already being used off-label for SLE, and several drugs in development will probably be available within the next few years.

### Anti-inflammatory targets: Anti-TNF, cytokines, toleragens and cell surface receptor inhibition

Many patients with RA who have SLE overlap disease have been treated with anti tumor necrosis factor products [23]. Only infliximab has been studied to any extent in pure SLE. Synovitis can be helped, but extra-articular manifestations may worsen and anti-DNA, anticardiolipin levels can appear or increase.

Anakinra (anti-IL-1Ra) is not effective for SLE, but tociluzumab (an anti-IL6) was quite potent in a 16-patient open label phase I trial at the National Institutes of Health [24]. An anti-interleukin (IL)-6 (CNTO 136; Johnson & Johnson, New Brunswick, NJ, USA/Centocor, Horsham, PA, USA) nephritis trial is due to start in late 2010. IL-10 can have favorable or unfavorable effects in SLE because of its pleomorphic properties; however, a favorable phase I safety trial of an anti-IL-10 (Schering, Berlin, Germany) is not likely to lead to further development because of its numerous contradictory actions. Promising strategies in murine SLE include inhibition of IL-12, -17, -18, -21 and -23, which may have translatable effects in humans.

La Jolla Pharmaceutics (La Jolla, CA, USA) LJP394 (Riquent) was an anti-anti-DNA B cell toleragen and edratide (TEVA, Petach Tikva, Isreal) a toleragen to the anti-16/6 anti-DNA idiotype [25,26]. Both were safe in trials involving hundreds of patients, but neither was effective enough to warrant further investigation. Laquinomod ( TEVA) has been tested in over 3,000 patients with multiple sclerosis and inflammatory bowel disease and appears to shift Th1 to Th2. An arthritis and nephritis trial was begun in late 2010. Lupuzor (Cephalon, Frazer, PA, USA) is a splicosomal peptide with U1 snRNP that promotes tolerance by preventing the proliferation of CD4+ T cells, as well as promoting secretion of IL-10 and decreasing anti-DNA in a European study. A phase IIb study is in progress.

Syk kinase inhibits intracellular kinases, and its clinical efficacy was demonstrated with R788 (Rigel, South San Francisco, CA, USA) in phase III RA trials [27]. There are plans to study this agent in SLE. Sirolimus (rapamycin) binds the regulatory kinase mTOR and is used for renal transplant rejection prevention. Many SLE patients with transplants currently take this agent, and a phase II trial is in progress.

### Innate immunity, including complement

Monoclonal antibodies to C5a were studied and shown to be safe in a phase I trial a decade ago with eculizumab (Solaris/Alexion, Cheshire, CT, USA), an agent now available for paroxysmal nocturnal hemoglobinuria [28]. A newer preparation from Novo Nordisk (Novo Nordisk, Bagsvaard, Denmark). had its SLE trial halted due to concerns relating to neutropenia in control patients.

Toll-like receptors (TLRs)-7 and -9 in immature dendritic cells are activated by complexes of self-protein and RNA or DNA. These complexes are normally rapidly cleared but accumulate in SLE because of clearance defects. TLR-7 and -9 activation induces secretion of interferons and promotes inflammation. Antimalarial drugs target TLR-7 and -9, and the development of a small oral molecule with similar actions has generated great interest from several companies (for example, ESAI (Woodcliff Lake, NJ, USA), Coley (Dusseldorff, Germany)/Xiphon (New Castle, Delaware, USA)/Pfizer (New York, NY, USA)). Medi-545 (sifalimumab; Medimmune (Gaithersburg, MDm USA)/Astra Zeneca (Wilmington, DE, USA)) and rontalizumab (Roche) can decrease the α-interferon signature within days by 90% by looking at protein and gene expression and clear lesions in serial skin biopsies in phase I studies [29,30] (Table 2). They are currently in phase III trials. NNC0152 (Novo Nordisk) and Neovasc are further behind in development, as is an agent which targets γ-interferon (AMG 811; Amgen).

## Summary and Conclusions

Providing industry with a guidance document has revolutionized SLE clinical trial development. It is likely that the first SLE drug to be approved in over 50 years will be marketed in 2011. Numerous other promising approaches are being considered that will improve the morbidity, mortality and quality of life of patients with SLE. These agents may decrease or make unnecessary the use of toxic agents such as steroids or immune suppressive agents. The above approaches will influence mild and organ-threatening disease, as well as having niche properties translatable into clinically relevant outcomes.

## Competing interests

Dr Wallace has performed clinical trials and/or consulted for Biogen, Amgen, Bristol-Myers Squibb, Roche, Cephalon, UCB, Merck/Serono, Novo Nordisk, Alexion, Centocor, Esai, TEVA, Rigel, Human Genome Sciences and Lilly.

## Pre-publication history

The pre-publication history for this paper can be accessed here:

http://www.biomedcentral.com/1741-7015/8/77/prepub
